# The LncRNA DUXAP10 Could Function as a Promising Oncogene in Human Cancer

**DOI:** 10.3389/fcell.2022.832388

**Published:** 2022-02-03

**Authors:** Junjie Zhao, Lixia Xu, Zihui Dong, Yize Zhang, Junhua Cao, Jie Yao, Jiyuan Xing

**Affiliations:** ^1^ Department of Pharmacy, The First Affiliated Hospital of Zhengzhou University, Zhengzhou, China; ^2^ Department of Infectious Diseases, The First Affiliated Hospital of Zhengzhou University, Zhengzhou, China; ^3^ Department of Plastic Surgery, The First Affiliated Hospital of Zhengzhou University, Zhengzhou, China; ^4^ Department of Ultrasound, The First Affiliated Hospital of Zhengzhou University, Zhengzhou, China

**Keywords:** DUXAP10, lncRNAs, cancer, function, clinical applications

## Abstract

Cancer is one of the most prevalent and deadliest diseases globally, with an increasing morbidity of approximately 14 million new cancer cases per year. Identifying novel diagnostic and prognostic biomarkers for cancers is important for developing cancer therapeutic strategies and lowering mortality rates. Long noncoding RNAs (lncRNAs) represent a group of noncoding RNAs of more than 200 nucleotides that have been shown to participate in the development of human cancers. The novel lncRNA DUXAP10 was newly reported to be abnormally overexpressed in several cancers and positively correlated with poor clinical characteristics of cancer patients. Multiple studies have found that DUXAP10 widely regulates vital biological functions related to the development and progression of cancers, including cell proliferation, apoptosis, invasion, migration, and stemness, through different molecular mechanisms. The aim of this review was to recapitulate current findings regarding the roles of DUXAP10 in cancers and evaluate the potential of DUXAP10 as a novel biomarker for cancer diagnosis, treatment, and prognostic assessment.

## Introduction

Despite significant advances in clinical diagnostic and treatment options, many diseases still have high mortality rates, high health care costs. And poor quality of life, especially cancer (Lee et al., 2018; Moliner et al., 2019; Sidney et al., 2019; Mulder et al., 2021; Su et al., 2021). Cancer (Mohsen et al., 2014; Christensen et al., 2018; Kannan et al., 2021; Mokgautsi et al., 2021) has become one of the most common causes of death worldwide, and the identification of cancer-related targets and relevant carcinogenesis mechanisms for patients at the early stage of the disease are urgently needed (Saha et al., 2019; Jiang et al., 2020; Brennan and Smith, 2021; Hua et al., 2021; Jones et al., 2021).

Long noncoding RNAs (Zhu et al., 2013; Rathinasamy and Velmurugan, 2018; Zheng et al., 2021; Ma et al., 2022) (lncRNAs) represent a special type of non-coding RNA with more than 200 nucleotides that are not translated into proteins. The aberrant expression of lncRNAs has been frequently observed in a variety of diseases, including human malignancies (Isin and Dalay, 2015; Bhan et al., 2017; Bian et al., 2021; Homayoonfal et al., 2021; Liu and Lei, 2021). Moreover, an increasing number of studies have shown that lncRNAs are involved in the pathogenesis and development of many cancers and are associated with different clinicopathological features (Wang et al., 2021b; Dong et al., 2021; Ma et al., 2021; Zhou et al., 2021). For example, overexpression of the lncRNA MCM3AP-AS1 (Wang et al., 2019b) has been shown to modulate hepatocellular carcinoma (HCC) occurrence and progression and is strongly correlated with advanced tumor grade and stage, large tumor size, and poor prognosis. Additionally, a high expression level of the lncRNA AK023391 (Huang et al., 2017) was found to exert a pivotal role in gastric cancer (GC) oncogenesis and development and was significantly linked to decreased survival rates. Functionally, lncRNAs have been demonstrated to frequently participate in the regulation of multiple crucial biological processes (Gao et al., 2020; Le et al., 2021; Yi et al., 2021; Zheng et al., 2021; Zhu et al., 2021), such as cell proliferation, apoptosis, and invasion. Given the complex functions of lncRNAs in cancers, lncRNAs exhibit tremendous potential for use in tumor diagnosis, prognosis, and treatment.

Recently, the pseudogene lncRNA DUXAP10 (DUXAP10) (Booth HA, and Holland PW., 2007; Zhu et al., 2018), localized on chromosome 14q11.2, was found to be overexpressed in multiple cancers, including HCC, bladder cancer (BC), non-small-cell lung cancer (NSCLC), glioma, renal cell carcinoma (RCC), papillary thyroid carcinoma (PTC), prostate cancer (PCa), chronic myelogenous leukemia (CML), ovarian cancer (OC), pancreatic cancer (PC), GC, colorectal cancer (CRC), esophageal squamous cell carcinoma (ESCC) and oral squamous cell carcinoma (OSCC). Its aberrant expression level was predominantly in line with many poor clinical characteristics. Studies of the biological functions of DUXAP10 showed that DUXAP10 can exhibit protumor effects on regulating cell processes, such as cell proliferation, apoptosis, migration, and invasion, by targeting specific genes or through a variety of different specific pathways. Therefore, the above evidence implicates the high potential of DUXAP10 as a biomarker for cancer diagnosis prognosis and therapy.

In this review, we summarize the roles of DUXAP10 in different cancers, including dysregulated expression, related clinical characteristics, biological functions underlying molecular mechanisms, and potential clinical applications.

## The Role of the LncRNA DUXAP10 in Different Cancers

Several studies have reported that DUXAP10 is aberrantly expressed in numerous human cancers, such as HCC, BC, NSCLC, glioma, RCC, PTC, PCa, CML, OC, PC, GC, CRC, ESCC, and OSCC. In addition, it was demonstrated that a high DUXAP10 expression level was positively related to advanced clinicopathological features (Table 1). The diverse regulatory functions and underlying mechanisms of DUXAP10 during tumor progression are shown in Table 2.

**TABLE 1:** DUXAP10 expression and clinicopathological features in cancers.

Disease type	Expression	Clinical characteristics	Refs
liver cancer	upregulation	overall survival rate and progression-free survival rate	(Zhu et al., 2018; Han et al., 2019; Sun et al., 2019)
kidney cancer	upregulation	male sex, tumor size, TNM stage, lymph node metastasis, pathologic stage, and overall survival rate	Chen et al. (2020)
lung cancer	upregulation	tumor size, pathological stage, lymph node metastasis, overall survival rate, relapse-free survival rate, and poor prognosis	(Wei et al., 2017; Lin et al., 2021)
glioma	upregulation		Wu et al. (2021)
thyroid carcinoma	upregulation		Li et al. (2020)
prostate cancer	upregulation		Wang et al. (2019a)
chronic myelogenous leukemia	upregulation	clinical stage	Yao et al. (2018)
ovarian cancer	upregulation	tumor size and FIGO stage	Zhang et al. (2018)
gastric cancer	upregulation	pathological stage, lymph node metastasis, and poor prognosis	Xu et al. (2018)
pancreatic cancer	upregulation	TNM stage, lymph node metastasis, and poor prognosis	Lian et al. (2018)
bladder cancer	upregulation		Lv et al. (2018)
colorectal cancer	upregulation	pathological stage, tumor size, lymph node metastasis, and poor prognosis	Lian et al. (2017)
esophageal squamous cell carcinoma	upregulation	TNM stage, lymph node metastasis, and survival time	Wang et al. (2018)

**TABLE 2:** Functions and mechanisms of DUXAP10 in cancers.

Disease type	Cell lines	Related mechanisms		Functions	Refs
		Molecule	Pathway		
liver cancer	Hep3B, Hep G2, SMMC7721, HuH7, MHCC-97L, MHCC-97H, HCC-LM, and SK-Hep-1	microRNA-1914, and GPR39	PI3K/AKT/mTOR pathway, and Wnt/β-catenin pathway	cell cycle, colony formation, proliferation, epithelial-mesenchymal transition, metastasis, and apoptosis	(Zhu et al., 2018; Han et al., 2019; Sun et al., 2019)
kidney cancer	786-O and A498			cell cycle, proliferation, apoptosis, migration, and invasion	Chen et al. (2020)
lung cancer	A549, H1975, SPC-A1, H1299, and BEAS-2B	Cd, Pax6, GLI1, LSD1, LATS2, and RRAD	Hedgehog pathway	cell cycle, proliferation, migration and invasion, and cancer stem cell transformation	(Wei et al., 2017; Lin et al., 2021)
glioma	HS683, U251, U373, U87, T98G LN-319 and SW1783	HuR, CD133, Oct4, and Sox12		cell stemness	Wu et al. (2021)
thyroid carcinoma	TPC-1, BCPAP, K1, and IHH-4		Akt/mTOR pathway	cell proliferation, apoptosis, invasion, and migration	Li et al. (2020)
prostate cancer	PC3, 22RV1, and DU145			cell cycle, proliferation, and metastasis	Wang et al. (2019a)
chronic myelogenous leukemia	THP-1, KG-1, and K562	PTEN		cell proliferation, apoptosis, and cell cycle	Yao et al. (2018)
ovarian cancer	HO8910 and A2780			cell proliferation	Zhang et al. (2018)
gastric cancer	BGC823, SGC7901, MGC803, AGS, HGC27, and MKN45	PRC2, LSD1, HuR, KLF2, LATS1, and *β*-catenin		cell proliferation, cell cycle, invasion, and migration	Xu et al. (2018)
pancreatic cancer	AsPC-1, BxPC-3, and PANC-1	EZH2, and LSD1		cell cycle, proliferation, and apoptosis	Lian et al. (2018)
bladder cancer	5,637, T24, E-j, TCCSUP, UM-UC-3, and RT4		PI3K/Akt/mTOR pathway	cell cycle, proliferation, and apoptosis	Lv et al. (2018)
colorectal cancer	HCT116, SW620, and SW480	LSD1, PTEN, and p21		cell proliferation, apoptosis, and cell cycle	Lian et al. (2017)
esophageal squamous cell carcinoma	KYSE30, KYSE510, KYSE180, and KYSE150	EZH2, and p21		cell cycle, proliferation, metastasis, and apoptosis	Wang et al. (2018)

We next discuss the DUXAP10 expression level, relevant clinical characteristics, and biological functions in different cancer types.

### Liver Cancer

Liver cancer is one of the leading causes of cancer death worldwide (Sun et al., 2013; Zhao et al., 2015; Kido et al., 2020). Hepatocellular carcinoma (HCC) is the most common type of primary liver cancer (Ahn et al., 2018; Xu et al., 2021). Numerous studies have recently revealed that DUXAP10 was overexpressed in HCC cell lines (including Hep3B, HepG2, SMMC7721, HuH7, MHCC-97L, MHCC-97H, HCC-LM, and SK-Hep-1 cells) and tissues (Zhu et al., 2018; Han et al., 2019; Sun et al., 2019). And its level was positively related to the severity of HCC, higher DUXAP10 expression was observed in advanced HCC patients. In addition, a high expression level of DUXAP10 was significantly correlated with poor overall survival (OS) and progression-free survival (PFS) rates (Han et al., 2019). It has also been proven to exert pivotal pro-oncogenic functions in the regulation of cell cycle progression, colony formation, proliferation, epithelial-mesenchymal transition (EMT), invasion, migration, and cell apoptosis in SMMC-7721, Hep G2, Hep3B, and MHCC-97L cells.

### Kidney Cancer

Renal cell carcinoma (RCC), arising from the renal epithelium (Wang et al., 2020b; Singh et al., 2020), is the most common kidney tumor (Wang et al., 2017; Chen et al., 2018). High expression of DUXAP10 was observed in 18 RCC specimens collected from the Urology Department of Peking University Shougang Hospital and 786-O and A498 cell lines in comparison with adjacent normal tissues and normal kidney epithelial cells (HKCs). Its level was positively correlated with male sex, tumor size, TNM stage, lymph node metastasis, and advanced pathologic stage. Additionally, Kaplan–Meier analysis further verified the strong link between DUXAP10 and poor overall patient survival. DUXAP10 has been proven to have pro-oncogenic functions in the regulation of cell proliferation, cell cycle transition to the S phase, cell apoptosis, cell migration, and invasion.

### Lung Cancer

Lung cancer (Molina et al., 2008; Zhang et al., 2019; Chen et al., 2021b) remains the leading cause of cancer-related mortality worldwide, with approximately 1.8 million (Chen et al., 2021a; Sung et al., 2021) deaths per year. While there has been a modest improvement in lung cancer (Sławińska-Brych et al., 2021) OS in recent decades, further studies are needed to improve patients’ clinical outcomes. Several studies have reported that DUXAP10 is significantly upregulated in 93 human cancer tissues obtained from The First and Second Affiliated Hospital of Nanjing Medical University, non-small-cell lung cancer (NSCLC) cell lines (A549, H1975, SPC-A1, and H1299 cells) and the chronic cadmium (Cd)-induced human bronchial epithelial BEAS-2B cell line. A high level of DUXAP10 expression in lung cancer patients was linked to not only lower OS and relapse-free survival (RFS) rates but also larger tumor sizes, advanced tumor stages, lymph node metastasis, and even poor prognosis (Wei et al., 2017; Lin et al., 2021). Moreover, *in vitro* functional assays and *in vivo* tumor models have demonstrated that DUXAP10 is mainly implicated in eliciting the transformation of Cd-exposed (Cd-T) cells to cancer stem cells (CSCs), promoting the cell cycle progression, proliferation, migration, and invasion of A549 or H1975 cells, and thus accelerating tumorigenesis and progression in lung cancer.

### Glioma

Gliomas are the most common primary malignancy of the central nervous system (Briançon-Marjollet et al., 2010; Deluche et al., 2019; Shi et al., 2020a). DUXAP10 has been indicated to be highly expressed in glioma cell lines (HS683, U251, U373, U87, T98G LN-319, and SW1783 cells) and tissues gained from patients under surgery at the First Affiliated Hospital of Jinan University. Notably, it was found that DUXAP10 (Wu et al., 2021) was involved in facilitating the stem cell-like properties of glioma U251 and T98G cells by increasing the expression of Sox2, CD133, Oct4 stemness markers, the ability of tumorsphere formation, and the activity of ALDH, which closely aligns with the CSC induction mentioned in the above chronic Cd exposure study on lung cancer.

### Thyroid Carcinoma

Thyroid carcinoma can be divided into four types: papillary thyroid carcinoma (PTC), follicular thyroid carcinoma, medullary thyroid carcinoma, and anaplastic thyroid carcinoma (Wen et al., 2019; Zhang et al., 2021). PTC is the most common type of thyroid carcinoma (Li et al., 2018; Hu et al., 2019; Wang et al., 2021a). DUXAP10 was first confirmed by Li et al. (2020) to be highly expressed in PTC tissues and TPC-1, BCPAP, K1, and IHH-4 cells compared with adjacent normal thyroid tissues. In addition, DUXAP10 has been demonstrated to contribute to protumorigenic effects by promoting BCPAP and K1 (Li et al., 2020) cell proliferation and invasion in addition to inhibiting cell apoptosis.

### Prostate Cancer

Prostate cancer (Chang et al., 2014; Siegel et al., 2021) (PCa) is one of the most frequently diagnosed malignancies in the male genitourinary system. In PCa (Luo et al., 2019; Shang et al., 2019; Wen et al., 2020), lncRNAs have been found to play increasingly vital roles in tumorigenesis and development in recent years. Previously, X-F Wang indicated that DUXAP10 was highly expressed in PCa tissues and PC3, 22RV1, and DU145 cell lines. Downregulation of DUXAP10 was able to suppress tumor development by inactivating the processes of cell cycle progression, cell proliferation, and metastasis in PC3 and DU145 cells (Wang et al., 2019b).

### Chronic Myelogenous Leukemia

DUXAP10 (Yao et al., 2018) was upregulated in chronic myelogenous leukemia (CML) THP-1, KG-1, and K562 cells, and its expression level was observed to gradually increase in response to clinical upstaging of CML (chronic phase, acceleration phase, and blast phase). Furthermore, *in vitro* functional assays showed that knockdown of DUXAP10 notably weakened cell proliferation and enhanced cell apoptosis and cell cycle arrest in K652 and KG-1 cells.

### Ovarian Cancer

Ovarian cancer (OC) has the highest mortality rate among gynecological cancers (Chen et al., 2013; Gurunathan et al., 2019; Yu et al., 2019). Studies have indicated that DUXAP10 (Zhang et al., 2018) is upregulated in OC tissues and cell lines. A high expression level of DUXAP10 was remarkably connected with tumor size and the FIGO stage. More importantly, DUXAP10 was involved in the momentous modulation of tumor progression by stimulating the proliferation of HO8910 and A2780 cells.

### Gastric Cancer

Gastric cancer (GC) is the fourth most common cancer and the third most common cause of cancer-related death worldwide (Sun et al., 2017; Zhao et al., 2017; Shin et al., 2021). DUXAP10 has been recently reported to show higher expression in GC (Xu et al., 2018) tissues and cell lines (including BGC823, SGC7901, MGC803, AGS, HGC27, and MKN45 cells) and was strongly correlated with deteriorating pathological stage, lymph node metastasis and even worse prognosis. In AGS, BGC823, SGC7901, and MGC803 cell lines, DUXAP10 was found to exert cancer-promoting functions through the activation of cell proliferation, cell cycle progression, invasion, and migration. In an *in vivo* BALB/c nude mouse tumor formation study, the mice exhibited larger tumor weights and sizes, which further verified the tumorigenic ability of DUXAP10.

### Pancreatic Cancer

In recent years, it has been found that DUXAP10 is excessively expressed in pancreatic (Lian et al., 2018) cancer (PC) tissues and cell lines (AsPC-1, BxPC-3, and PANC-1 cells), and a close correlation was observed between the overexpression of DUXAP10 and unfavorable clinicopathological characteristics, such as poor prognosis, aggressive TNM stage and lymph node metastasis. Functional analyses in BxPC-3 and PANC-1 cell lines provided powerful evidence that DUXAP10 accelerated cell cycle progression and proliferation and suppressed cell apoptosis. Additionally, *in vivo* xenograft tumor model experiments validated the tumor-promoting role of DUXAP10 in accelerating tumor growth and increasing tumor volumes.

### Bladder Cancer

Bladder cancer (BC) is the most common cancer of the urinary tract (Gan et al., 2016; Loras et al., 2019). Lv et al. (2018) proposed for the first time that DUXAP10 was overexpressed in BC tissues and 5,637, T24, E-j, TCCSUP, UM-UC-3, and RT4 cells. Additionally, DUXAP10 contributed to cancer progression through its involvement in several cellular functions in T24 and 5,637 cells, including cell cycle progression, proliferation, and apoptosis.

### Colorectal Cancer

Over the past decade, DUXAP10 was highly expressed in colorectal cancer (CRC) (Lian et al., 2017) tissues and HCT116, SW620, and SW480 cell lines. DUXAP10 was also found to be positively correlated with advanced pathological stages, larger tumor sizes, lymph node metastasis, and poor prognosis. Knocking down DUXAP10 attenuated the proliferative ability, accelerated the apoptotic process, and blocked the cell cycle progression of HCT116 and SW480 cells. Subsequently, the increased tumor volumes and weights in experimental tumor models further demonstrated the carcinogenicity of DUXAP10.

### Esophageal Squamous Cell Carcinoma

Esophageal squamous cell carcinoma (ESCC) (Pan et al., 2019; Wang et al., 2020a; Li et al., 2021) is the predominant subtype of esophageal carcinoma, with a poor 5-years survival (De Angelis et al., 2014) rate of less than 21%. DUXAP10 has been found to be expressed at high levels in ESCC tissues and cells (KYSE30, KYSE510, KYSE180, and KYSE150 cells), whereas overexpression of DUXAP10 was closely correlated with short survival time, TNM stage, and lymph node metastasis. In addition, DUXAP10 essentially participates in ESCC development and progression by enhancing cell proliferation and metastasis, accelerating cell cycle progression, and hindering cell apoptosis in KYSE30 and KYSE180 cells.

### Head and Neck Squamous Cell Carcinoma

Head and neck squamous cell carcinoma (HNSCC) (Meehan et al., 2020) represents a heterogeneous mucosal malignancy derived from the oral, oropharyngeal, hypopharyngeal, and laryngeal cavities. Oral squamous cell carcinoma (Feng et al., 2017; Jia et al., 2020) (OSCC) and oropharyngeal squamous cell carcinoma (OPSCC) are the most frequent types of HNSCC, accounting for 377,713 new cases and 177,757 (Sung et al., 2021) deaths worldwide. Recent studies have also demonstrated that DUXAP10 is differentially expressed in OSCC and OPSCC tissues.

## Regulatory Mechanisms of the LncRNA DUXAP10

**FIGURE 1:**
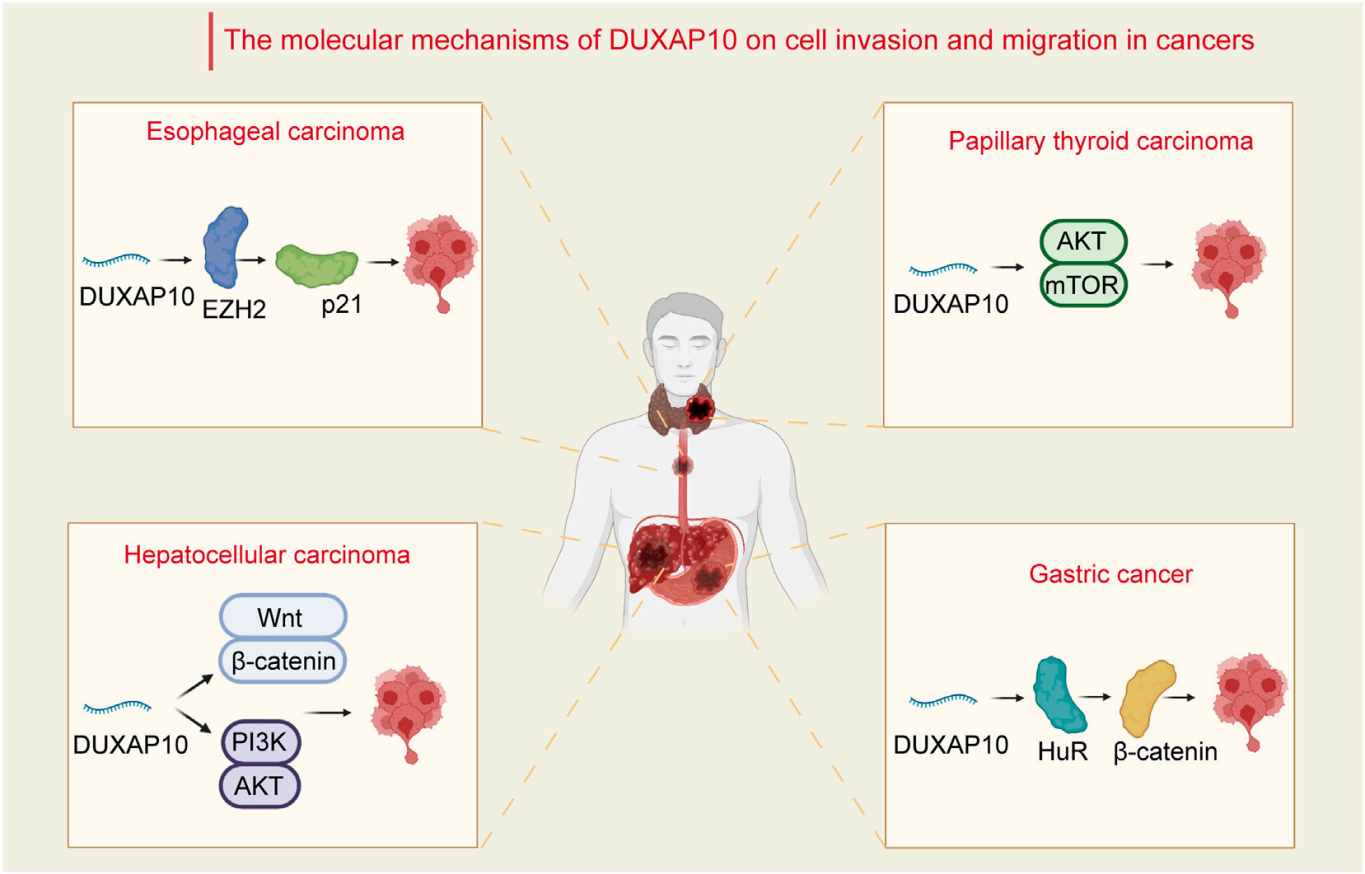
Relevant molecular mechanisms of DUXAP10 in the process of cell invasion and migration in cancers. In hepatocellular carcinoma, DUXAP10 enhances cell invasion and migration through the Wnt/*β*-catenin and PI3K/Akt signaling pathways. In papillary thyroid carcinoma, DUXAP10 mediates the activity of the Akt/mTOR pathway to regulate cancer cell invasion and migration. In gastric cancer, DUXAP10 is involved in the regulation of invasion and migration via combination with the RNA-binding protein HuR and subsequently increases the stability of *β*-catenin. In esophageal carcinoma, DUXAP10 regulates cell metastasis by binding with EZH2 and inhibiting p21 expression.

As a newfound oncogene, DUXAP10 has been reported to be widely involved in the mediation of several crucial biological processes, such as cell proliferation, apoptosis, and metastasis, in diverse cancer types. Here, we mainly provide a current understanding of the major biological functions and corresponding molecular mechanisms of DUXAP10 (Figure 1).

### Cell Proliferation and Apoptosis

**FIGURE 2:**
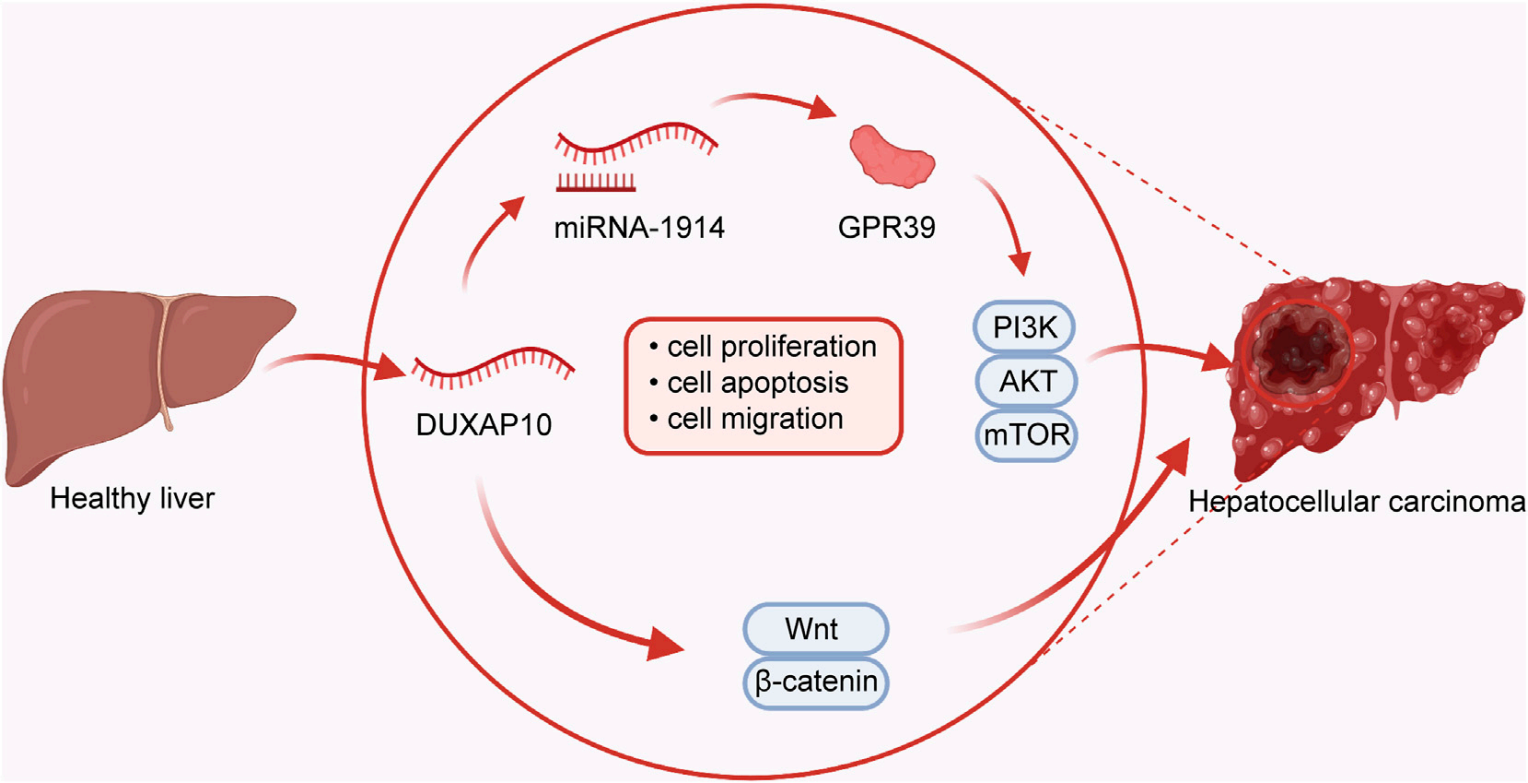
In hepatocellular carcinoma, DUXAP10 plays an oncogenic role in regulating the processes of cell proliferation, apoptosis, and migration by combining with microRNA-1914 and further activating the GPR39-dependent PI3K/AKT/mTOR pathway or Wnt/*β*-catenin pathway.

Cell proliferation and apoptosis are fundamental for normal cell growth and development. Abnormal cell growth is a key marker for cancer (Shi et al., 2020a; Go et al., 2021; Hu et al., 2021). It has been shown that microRNA-1914 could increase the effect of DUXAP10 on cell proliferation and apoptosis through activation of the GPR39-mediated PI3K/AKT/mTOR pathway in HCC Hep3B and MHCC-97L (Sun et al., 2019) cells (Figure 2). In addition, the proliferative mechanism of DUXAP10 in HCC SMMC-7721 and HepG2 (Han et al., 2019) cells was observed via the Wnt/β-catenin and PI3K/Akt signaling pathways. Likewise, one study has shown that DUXAP10 can enhance cell cycle progression and subsequent cell proliferation in NSCLC (Wei et al., 2017) A549 or H1975 cells by specifically binding to LSD1 and increasing the levels of LATS2 and RRAD. In PTC (Li et al., 2020), there was experimental evidence that DUXAP10 restrained cell apoptosis via inactivation of Caspase-3, in turn resulting in the promotion of proliferative ability in BCPAP and K1 cells. Moreover, DUXAP10 was found to facilitate cell proliferation and inhibit the apoptosis of CML (Yao et al., 2018) K652 and KG-1 cells by inhibiting PTEN expression. Functional studies in GC (Xu et al., 2018) BGC823, SGC7901, and MGC803 cells also suggest a critical regulatory role for DUXAP10 in cell proliferation by directly interacting with PRC2 and LSD1, thus repressing the expression of LATS1. Similarly, DUXAP10 has been indicated to induce PC BxPC-3 and PANC-1 cell proliferation and suppress apoptosis through combination with the RNA-binding proteins EZH2 and LSD1. Mechanistic research in T24 and 5,637 (Lv et al., 2018) BC cells has also proven that DUXAP10 regulates cell proliferation and apoptosis by modulating the PI3K/Akt/mTOR signaling pathway. In CRC (Lian et al., 2017), DUXAP10 regulates the expression of p21 and phosphatase and tensin homolog (PTEN) by binding to the histone demethylase lysine-specific demethylase 1 (LSD1), enhancing CRC cell proliferation and reducing apoptosis. As shown in KYSE30 and KYSE180 ESCC (Wang et al., 2018) cells, DUXAP10 has been similarly confirmed to modulate the processes of cell proliferation and apoptosis by negatively modulating p21 expression by interacting with zeste homolog 2 (EZH2).

### Cell Invasion and Migration

Cancer metastasis remains one of the biggest challenges in cancer therapy (Gou et al., 2020; Wang et al., 2021c). Migration and invasion are prerequisites for cancer cell metastasis (Zacharias et al., 2018). The mechanisms of cell migration and invasion have been a focus of research. Current studies have revealed that DUXAP10 mainly affects the processes of tumor cell EMT, thus enhancing tumor cell migratory properties in several cancers. By modulating the Wnt/β-catenin and PI3K/Akt signaling pathways, DUXAP10 has been demonstrated to influence the invasion and migration of HCC (Han et al., 2019) SMMC-7721 and HepG2 cells through the regulation of EMT. A previous study of PTC also demonstrated that DUXAP10 was involved in increasing cell invasion and migration and regulating EMT via activation of the Akt/mTOR (Li et al., 2020) pathway. Additionally, DUXAP10 was found to play a crucial role in GC (Xu et al., 2018) cell invasiveness and migration by interacting with the RNA-binding protein HuR and subsequently stabilizing *β*-catenin mRNA. It was also demonstrated that in ESCC KYSE30 and KYSE180 cells, DUXAP10 regulated cell metastasis by binding with EZH2 and downregulating p21 (Wang et al., 2018) expression.

### Cell Stemness

The CSC hypothesis suggests that CSCs (Tang, 2012; Eun et al., 2017) are a subpopulation of tumor cells with the characteristics of powerful self-renewal and aberrant differentiation potential. Interestingly, an emerging role of DUXAP10 in promoting the transition of CSCs has been observed, which is essential for the tumorigenicity of cancer cells.

4 A recent study using a post-chronic Cd-exposed human bronchial epithelium BEAS-2B cell model demonstrated that DUXAP10 induces CSC-like (Lin et al., 2021) properties by improving GLI1 protein stability and ultimately regulating the Hedgehog signaling pathway. In Cd-exposed transformed cells, Hedgehog signaling pathway was activated and subsequently involved in mediating CSC-like characteristics. At the same time, Pax6 was upregulated and significantly increase the duxap10 level and CSC like characteristics. It was also found that DUXAP10 promotes the stemness of glioma (Wu et al., 2021) U251 and T98G cells by binding to HuR and thus upregulating the expression of Sox12. DUXAP10 knockdown remarkably reduced the activity of ALDH and the expression of stemness markers (Sox2, CD133, Oct4) in glioma cells. Thus, these results have shown DUXAP10 had vital effects on glioma cell stemness. Promising Clinical Applications of DUXAP10.

Accumulating studies have shown that dysregulated lncRNA (Bhan et al., 2017; Gao et al., 2021; Tan et al., 2021) expression contributes to the development of tumors and can be used as a promising marker for lncRNA-based applications in cancer management. Aberrantly expressed DUXAP10 was recently revealed to be involved in a wide range of biological functions and pathological characteristics, and its vital clinical applications in several cancer types are valuable for clinical diagnosis, prognosis, and treatment management. In this section, we describe the meaningful medicinal applications of DUXAP10 in numerous tumor types.

### DUXAP10 as a Diagnostic and Prognostic Biomarker

It is now widely accepted that the early diagnosis (An et al., 2021; Kannan et al., 2021) of tumors is essential for achieving a better prognosis and a lower mortality rate. Accurate diagnostic biomarkers (Ott et al., 2009; Wardle et al., 2015; Dragani et al., 2020) for detecting early-stage tumors are of great clinical significance. An increasing number of oncology studies have reported that the overexpression of DUXAP10 in diverse tumor tissues (such as NSCLC (Wei et al., 2017), glioma (Wu et al., 2021), and ESCC (Wang et al., 2018)) could be used to distinguish normal from tumor tissues, making it highly promising for the early diagnosis of tumors.

In addition, high DUXAP10 expression was closely associated with more advanced tumor stage or grade, earlier lymph node metastasis, and unfavorable OS, PFS, and RFS rates, which provides powerful evidence for the prognostic ability of DUXAP10 in various cancers, such as HCC (Zhu et al., 2018), NSCLC (Wei et al., 2017), RCC (Chen et al., 2020), OC (Zhang et al., 2018), GC (Xu et al., 2018), CRC (Lian et al., 2017), and ESCC (Wang et al., 2018). Therefore, DUXAP10 in combination with relevant clinicopathological features can function as an independent prognostic indicator in diverse cancer types.

### DUXAP10 as a Treatment Target

In recent decades, several reports have shown that lncRNAs (Yin et al., 2018; Diniz et al., 2021; Homayoonfal et al., 2021) play important roles in tumor progression and could be biomarkers for clinical treatments. Increasing studies have demonstrated that DUXAP10 is involved in the tumorigenesis and development of tumors through the modulation of diverse cellular processes, comprising cell colony formation, cell cycle progression, cell proliferation, apoptosis, metastasis, and even CSC-like properties. Moreover, numerous molecular mechanism experiments have confirmed that DUXAP10 plays a tumor promoter role by regulating key target gene activity and affecting multiple important signaling pathways, making it a possible molecular factor that can be used as a therapeutic target in HCC (Han et al., 2019; Sun et al., 2019), NSCLC (Wei et al., 2017), glioma (Wu et al., 2021), RCC (Chen et al., 2020), PTC (Li et al., 2020), OC (Zhang et al., 2018), GC (Xu et al., 2018), PC (Lian et al., 2018), BC (Lv et al., 2018), CRC (Lian et al., 2017) and ESCC (Wang et al., 2018). Therefore, a further in-depth understanding of the pro-oncogenic mechanisms of DUXAP10 for cancer treatment is needed.

## Conclusion

With the advent of novel genomic approaches as well as technological breakthroughs, an unprecedented understanding of the development of cancer has provided novel insight for establishing more effective cancer management strategies. Mounting evidence indicates that DUXAP10 is abnormally highly expressed in a variety of cancers, including HCC, lung cancer, glioma, RCC, PTC, PCa, CML, OC, GC, PC, BC, CRC, and ESCC. The higher expression of DUXAP10 in tumor tissues compared with adjacent normal tissues indicates the diagnostic potential of using DUXAP10 to successfully distinguish cancerous tissues from normal tissues. However, the detection of DUXAP10 in tissue is an invasive and complicated method, and further studies are needed to search for possible noninvasive methods for the diagnosis of cancers using DUXAP10.

In addition, DUXAP10 overexpression was positively correlated with the adverse clinicopathological features and aggressive outcomes of several cancer types, including tumor size, TNM staging, histological grading, lymph node metastasis, and survival rates. These associations revealed the potential for DUXAP10 to be used as a prognostic biomarker to predict cancer prognosis and provide guiding recommendations for the future treatment of tumors. More importantly, DUXAP10 has been found to play an oncogenic role and participate in the biological processes of cellular proliferation, apoptosis, invasion, migration, and stemness through the regulation of various target genes or signaling pathways. Thus, the molecular mechanisms of DUXAP10 involved in tumor progression should be further explored, contributing to new hopes in tumor treatment.

## References

[B1] Ahn S.-M. Haq F. Park I. Nault J.-C. Zucman-Rossi J. Yu E. (2018). The Clinical Implications of G1-G6 Transcriptomic Signature and 5-gene Score in Korean Patients with Hepatocellular Carcinoma. BMC Cancer 18, 571. 10.1186/s12885-018-4192-1 29776391 PMC5960090

[B2] An L. Yu R. Han Y. Zhou Z. (2021). Decoding the Intercellular Communication Network during Tumorigenesis. Cancer Biol. Med. 18. 10.20892/j.issn.2095-3941.2021.0558 PMC8958884 34783465

[B3] Bhan A. Soleimani M. Mandal S. S. (2017). Long Noncoding RNA and Cancer: a New Paradigm. Cancer Res. 77, 3965–3981. 10.1158/0008-5472.can-16-2634 28701486 PMC8330958

[B4] Bian Z. Zhou M. Cui K. Yang F. Cao Y. Sun S. (2021). SNHG17 Promotes Colorectal Tumorigenesis and Metastasis via Regulating Trim23-PES1 axis and miR-339-5p-FOSL2-SNHG17 Positive Feedback Loop. J. Exp. Clin. Cancer Res. 40, 360. 10.1186/s13046-021-02162-8 34782005 PMC8591805

[B5] Booth H. A. F. Holland P. W. H. (2007). Annotation, Nomenclature and Evolution of Four Novel Homeobox Genes Expressed in the Human Germ Line. Gene 387, 7–14. 10.1016/j.gene.2006.07.034 17005330

[B6] Brennan P. Davey-Smith G. (2021). Identifying Novel Causes of Cancers to Enhance Cancer Prevention: New Strategies Are Needed. J. Natl. Cancer Inst. 10.1093/jnci/djab204 PMC8902436 34743211

[B7] Briançon-Marjollet A. Balenci L. Fernandez M. Estève F. Honnorat J. Farion R. (2010). NG2-expressing Glial Precursor Cells Are a New Potential Oligodendroglioma Cell Initiating Population in N -ethyl- N -Nitrosourea-Induced Gliomagenesis. Carcinogenesis 31, 1718–1725. 10.1093/carcin/bgq154 20651032 PMC3308186

[B8] Chang A. J. Autio K. A. Roach M. 3rd Scher H. I. (2014). High-risk Prostate Cancer-Classification and Therapy. Nat. Rev. Clin. Oncol. 11, 308–323. 10.1038/nrclinonc.2014.68 24840073 PMC4508854

[B9] Chen H. Landen C. N. Li Y. Alvarez R. D. Tollefsbol T. O. (2013). Enhancement of Cisplatin-Mediated Apoptosis in Ovarian Cancer Cells through Potentiating G2/M Arrest and P21 Upregulation by Combinatorial Epigallocatechin Gallate and Sulforaphane. J. Oncol. 2013, 1–9. 10.1155/2013/872957 PMC3588178 23476648

[B10] Chen J. Wang X. F. Qin Y. C. Gong Y. B. Wang L. Li N. C. (2020). Downregulation of Long Non-coding RNA DUXAP10 Inhibits Proliferation, Migration, and Invasion of Renal Cell Carcinoma. Eur. Rev. Med. Pharmacol. Sci. 24, 11041–11051. 10.26355/eurrev_202011_23589 33215419

[B11] Chen K.-C. Tsai S.-W. Zhang X. Zeng C. Yang H.-Y. (2021a). The Investigation of the Volatile Metabolites of Lung Cancer from the Microenvironment of Malignant Pleural Effusion. Sci. Rep. 11, 13585. 10.1038/s41598-021-93032-y 34193905 PMC8245642

[B12] Chen R.-L. Sun L.-L. Cao Y. Chen H.-R. Zhou J.-X. Gu C.-Y. (2021b). Adjuvant EGFR-TKIs for Patients with Resected EGFR-Mutant Non-small Cell Lung Cancer: a Meta-Analysis of 1,283 Patients. Front. Oncol. 11, 629394. 10.3389/fonc.2021.629394 33912453 PMC8071858

[B13] Chen Z.-y. Du Y. Wang L. Liu X.-h. Guo J. Weng X.-d. (2018). MiR-543 Promotes Cell Proliferation and Metastasis of Renal Cell Carcinoma by Targeting Dickkopf 1 through the Wnt/β-Catenin Signaling Pathway. J. Cancer 9, 3660–3668. 10.7150/jca.27124 30405834 PMC6216004

[B14] Christensen J. F. Simonsen C. Hojman P. (2018). Exercise Training in Cancer Control and Treatment. Compr. Physiol. 9, 165–205. 10.1002/cphy.c180016 30549018

[B15] De Angelis R. Sant M. Coleman M. P. Francisci S. Baili P. Pierannunzio D. (2014). Cancer Survival in Europe 1999-2007 by Country and Age: Results of EUROCARE-5-A Population-Based Study. Lancet Oncol. 15, 23–34. 10.1016/s1470-2045(13)70546-1 24314615

[B16] Deluche E. Bessette B. Durand S. Caire F. Rigau V. Robert S. (2019). *CHI3L1, NTRK2, 1p/19q* and *IDH* Status Predicts Prognosis in Glioma. Cancers 11, 544. 10.3390/cancers11040544 PMC6521129 30991699

[B17] Diniz T. P. da Costa W. L. Jr. Gomes C. C. de Jesus V. H. F. Felismino T. C. Torres S. M. (2021). Symptomatic Recurrence and Survival Outcomes after Curative Treatment of Gastric Cancer: Does Intensive Follow-Up Evaluation Improve Survival? Ann. Surg. Oncol. 29, 274–284. 10.1245/s10434-021-10724-5 34782973

[B18] Dong X. Pi Q. Yuemaierabola A. Guo W. Tian H. (2021). Silencing LINC00294 Restores Mitochondrial Function and Inhibits Apoptosis of Glioma Cells under Hypoxia via the miR-21-5p/CASKIN1/cAMP axis. Oxidative Med. Cell Longevity 2021, 1–21. 10.1155/2021/8240015 PMC8580631 34777696

[B19] Dragani T. A. Matarese V. Colombo F. (2020). Biomarkers for Early Cancer Diagnosis: Prospects for success through the Lens of Tumor Genetics. Bioessays 42, 1900122. 10.1002/bies.201900122 32128843

[B20] Eun K. Ham S. W. Kim H. (2017). Cancer Stem Cell Heterogeneity: Origin and New Perspectives on CSC Targeting. BMB Rep. 50, 117–125. 10.5483/bmbrep.2017.50.3.222 27998397 PMC5422023

[B21] Feng L. Houck J. R. Lohavanichbutr P. Chen C. (2017). Transcriptome Analysis Reveals Differentially Expressed lncRNAs between Oral Squamous Cell Carcinoma and Healthy Oral Mucosa. Oncotarget 8, 31521–31531. 10.18632/oncotarget.16358 28415559 PMC5458226

[B22] Gan X. Lin X. He R. Lin X. Wang H. Yan L. (20162016). Prognostic and Clinicopathological Significance of Downregulated P16 Expression in Patients with Bladder Cancer: a Systematic Review and Meta-Analysis. Dis. Markers 2016, 1–13. 10.1155/2016/5259602 PMC4854991 27199504

[B23] Gao F. Cai Y. Kapranov P. Xu D. (2020). Reverse-genetics Studies of lncRNAs-What We Have Learnt and Paths Forward. Genome Biol. 21, 93. 10.1186/s13059-020-01994-5 32290841 PMC7155256

[B24] Gao M. Liu L. Yang Y. Li M. Ma Q. Chang Z. (2021). LncRNA HCP5 Induces Gastric Cancer Cell Proliferation, Invasion, and EMT Processes through the miR-186-5p/WNT5A axis under Hypoxia. Front. Cel Dev. Biol. 9, 663654. 10.3389/fcell.2021.663654 PMC8226141 34178988

[B25] Go S. Kramer T. T. Verhoeven A. J. Oude Elferink R. P. J. Chang J.-C. (2021). The Extracellular Lactate-To-Pyruvate Ratio Modulates the Sensitivity to Oxidative Stress-Induced Apoptosis via the Cytosolic NADH/NAD+ Redox State. Apoptosis 26, 38–51. 10.1007/s10495-020-01648-8 33230593 PMC7902596

[B26] Gou X.-j. Bai H.-h. Liu L.-w. Chen H.-y. Shi Q. Chang L.-s. (2020). Asiatic Acid Interferes with Invasion and Proliferation of Breast Cancer Cells by Inhibiting WAVE3 Activation through PI3K/AKT Signaling Pathway. Biomed. Res. Int. 2020, 1–12. 10.1155/2020/1874387 PMC7035546 32104680

[B27] Guo J.-H. Li L.-Y. Ma W. Li C.-Y. Zhang S.-J. Zang C.-H. (2022). Neuroprotective Effects of Long Noncoding RNAs Involved in Ischemic Postconditioning after Ischemic Stroke. Neural Regen. Res. 17, 1299–1309. 10.4103/1673-5374.327346 34782575 PMC8643058

[B28] Gurunathan S. Qasim M. Park C. H. Arsalan Iqbal M. Yoo H. Hwang J. H. (2019). Cytotoxicity and Transcriptomic Analyses of Biogenic Palladium Nanoparticles in Human Ovarian Cancer Cells (SKOV3). Nanomaterials 9, 787. 10.3390/nano9050787 PMC6566439 31121951

[B29] Han K. Li C. Zhang X. Shang L. (2019). DUXAP10 Inhibition Attenuates the Proliferation and Metastasis of Hepatocellular Carcinoma Cells by Regulation of the Wnt/β-Catenin and PI3K/Akt Signaling Pathways. Biosci. Rep. 39, BSR20181457. 10.1042/bsr20181457 30996112 PMC6542759

[B30] Homayoonfal M. Asemi Z. Yousefi B. (2021). Targeting Long Non Coding RNA by Natural Products: Implications for Cancer Therapy. Crit. Rev. Food Sci. Nutr., 1–29. 10.1080/10408398.2021.2001785 34783279

[B31] Hu S. Liao Y. Zheng J. Gou L. Regmi A. Zafar M. I. (2019). In Silico integration Approach Reveals Key MicroRNAs and Their Target Genes in Follicular Thyroid Carcinoma. Biomed. Res. Int. 2019, 1–9. 10.1155/2019/2725192 PMC6458921 31032340

[B32] Hua Y. You R. Wang Z. Huang P. Lin M. Ouyang Y. (2021). Toripalimab Plus Intensity-Modulated Radiotherapy for Recurrent Nasopharyngeal Carcinoma: an Open-Label Single-Arm, Phase II Trial. J. Immunother. Cancer 9, e003290. 10.1136/jitc-2021-003290 34782428 PMC8593727

[B33] Huang Y. Zhang J. Hou L. Wang G. Liu H. Zhang R. (2017). LncRNA AK023391 Promotes Tumorigenesis and Invasion of Gastric Cancer through Activation of the PI3K/Akt Signaling Pathway. J. Exp. Clin. Cancer Res. 36, 194. 10.1186/s13046-017-0666-2 29282102 PMC5745957

[B34] Isin M. Dalay N. (2015). LncRNAs and Neoplasia. Clinica Chim. Acta 444, 280–288. 10.1016/j.cca.2015.02.046 25748036

[B35] Jia H. Wang X. Sun Z. (2020). Exploring the Long Noncoding RNAs-Based Biomarkers and Pathogenesis of Malignant Transformation from Dysplasia to Oral Squamous Cell Carcinoma by Bioinformatics Method. Eur. J. Cancer Prev. 29, 174–181. 10.1097/cej.0000000000000527 31343435 PMC7012364

[B36] Jiang Y. Liu L. Xiang Q. He X. Wang Y. Zhou D. (2020). SEPT9_v2, Frequently Silenced by Promoter Hypermethylation, Exerts Anti-tumor Functions through Inactivation of Wnt/β-Catenin Signaling Pathway via miR92b-3p/FZD10 in Nasopharyngeal Carcinoma Cells. Clin. Epigenet 12, 41. 10.1186/s13148-020-00833-5 PMC7059696 32138771

[B37] Jones D. Di Martino E. Bradley S. H. Essang B. Hemphill S. Wright J. M. (2021). Factors Affecting the Decision to Investigate Older Adults with Potential Cancer Symptoms: a Systematic Review. Br. J. Gen. Pract. 72, e1–e10. 10.3399/bjgp.2021.0257 34782315 PMC8597772

[B38] Juncheng Hu J. Tianci Wang T. Jin Xu J. Sanyun Wu S. Liyuan Wang L. Hexiu Su H. (2021). WEE1 Inhibition Induces Glutamine Addiction in T-Cell Acute Lymphoblastic Leukemia. haematol 106, 1816–1827. 10.3324/haematol.2019.231126 PMC8252940 31919076

[B39] Kannan A. Clouston D. Frydenberg M. Ilic D. Karim M. N. Evans S. M. (2021). Neuroendocrine Cells in Prostate Cancer Correlate with Poor Outcomes: a Systematic Review and Meta‐analysis. BJU Int. 10.1111/bju.15647 34784097

[B40] Kido T. Tabatabai Z. L. Chen X. Lau Y. F. C. (2020). Potential Dual Functional Roles of the Y‐linked RBMY in Hepatocarcinogenesis. Cancer Sci. 111, 2987–2999. 10.1111/cas.14506 32473614 PMC7419034

[B41] Le T. He X. Huang J. Liu S. Bai Y. Wu K. (2021). Knockdown of Long Noncoding RNA GAS5 Reduces Vascular Smooth Muscle Cell Apoptosis by Inactivating EZH2-Mediated RIG-I Signaling Pathway in Abdominal Aortic Aneurysm. J. Transl. Med. 19, 466. 10.1186/s12967-021-03023-w 34781960 PMC8594130

[B42] Lee J. M. Park D. Y. Yang L. Kim E.-J. Ahrberg C. D. Lee K.-B. (2018). Generation of Uniform-Sized Multicellular Tumor Spheroids Using Hydrogel Microwells for Advanced Drug Screening. Sci. Rep. 8, 17145. 10.1038/s41598-018-35216-7 30464248 PMC6249215

[B43] Li H. Weng J. Shi Y. Gu W. Mao Y. Wang Y. (2018). An Improved Deep Learning Approach for Detection of Thyroid Papillary Cancer in Ultrasound Images. Sci. Rep. 8, 6600. 10.1038/s41598-018-25005-7 29700427 PMC5920067

[B44] Li J. Jiang L. Liu Z. Li Y. Xu Y. Liu H. (2020). Oncogenic Pseudogene DUXAP10 Knockdown Suppresses Proliferation and Invasion and Induces Apoptosis of Papillary Thyroid Carcinoma Cells by Inhibition of Akt/mTOR Pathway. Clin. Exp. Pharmacol. Physiol. 47, 1473–1483. 10.1111/1440-1681.13310 32215944

[B45] Li X. Zhao L. Wei M. Lv J. Sun Y. Shen X. (2021). Serum Metabolomics Analysis for the Progression of Esophageal Squamous Cell Carcinoma. J. Cancer 12, 3190–3197. 10.7150/jca.54429 33976728 PMC8100812

[B46] Lian Y. Xiao C. Yan C. Chen D. Huang Q. Fan Y. (2018). Knockdown of Pseudogene Derived from lncRNA DUXAP10 Inhibits Cell Proliferation, Migration, Invasion, and Promotes Apoptosis in Pancreatic Cancer. J. Cel. Biochem. 119, 3671–3682. 10.1002/jcb.26578 29286182

[B47] Lian Y. Xu Y. Xiao C. Xia R. Gong H. Yang P. (2017). The Pseudogene Derived from Long Non-coding RNA DUXAP10 Promotes Colorectal Cancer Cell Growth through Epigenetically Silencing of P21 and PTEN. Sci. Rep. 7, 7312. 10.1038/s41598-017-07954-7 28779166 PMC5544748

[B48] Lin H.-P. Wang Z. Yang C. (2021). LncRNA DUXAP10 Upregulation and the Hedgehog Pathway Activation Are Critically Involved in Chronic Cadmium Exposure-Induced Cancer Stem Cell-like Property. Toxicol. Sci. 184, 33–45. 10.1093/toxsci/kfab099 34373904 PMC8677432

[B49] Liu Q. Lei C. (2021). LINC01232 Serves as a Novel Biomarker and Promotes Tumour Progression by Sponging miR-204-5p and Upregulating RAB22A in clear Cell Renal Cell Carcinoma. Ann. Med. 53, 2153–2164. 10.1080/07853890.2021.2001563 34783622 PMC8604453

[B50] Liu W. Zhao X. Chen Q. Li Y. Tang H. Liu X. (2015). Codelivery of Doxorubicin and Curcumin With lipid Nanoparticles Results in Improved Efficacy Of chemotherapy in Liver Cancer. Ijn 10, 257–270. 10.2147/ijn.s73322 25565818 PMC4284012

[B51] Loras A. Martínez-Bisbal M. C. Quintás G. Gil S. Martínez-Máñez R. Ruiz-Cerdá J. L. (2019). Urinary Metabolic Signatures Detect Recurrences in Non-muscle Invasive Bladder Cancer. Cancers 11, 914. 10.3390/cancers11070914 PMC6678457 31261883

[B52] Luo J. Wang K. Yeh S. Sun Y. Liang L. Xiao Y. (2019). LncRNA-p21 Alters the Antiandrogen Enzalutamide-Induced Prostate Cancer Neuroendocrine Differentiation via Modulating the EZH2/STAT3 Signaling. Nat. Commun. 10, 2571. 10.1038/s41467-019-09784-9 31189930 PMC6561926

[B53] Lv X.-Y. Ma L. Chen J.-F. Yu R. Li Y. Yan Z. J. (2018). Knockdown of DUXAP10 Inhibits Proliferation and Promotes Apoptosis in Bladder Cancer Cells via PI3K/Akt/mTOR Signaling Pathway. Int. J. Oncol. 52, 288–294. 10.3892/ijo.2017.4195 29115412

[B54] Ma B. Jiang H. Luo Y. Liao T. Xu W. Wang X. (2021). Tumor-infiltrating Immune-Related Long Non-coding RNAs Indicate Prognoses and Response to PD-1 Blockade in Head and Neck Squamous Cell Carcinoma. Front. Immunol. 12, 692079. 10.3389/fimmu.2021.692079 34737735 PMC8562720

[B55] Meehan K. Leslie C. Lucas M. Jacques A. Mirzai B. Lim J. (2020). Characterization of the Immune Profile of Oral Tongue Squamous Cell Carcinomas with Advancing Disease. Cancer Med. 9, 4791–4807. 10.1002/cam4.3106 32383556 PMC7333861

[B56] Mohsen H. Haddad P. Allam A. Hassan A. (2014). Patterns in Place of Cancer Death in the State of Qatar: a Population-Based Study. PLoS One 9, e109615. 10.1371/journal.pone.0109615 25536076 PMC4275179

[B57] Mokgautsi N. Wang Y.-C. Lawal B. Khedkar H. Sumitra M. R. Wu A. T. H. (2021). Network Pharmacological Analysis through a Bioinformatics Approach of Novel NSC765600 and NSC765691 Compounds as Potential Inhibitors of *CCND1/CDK4/PLK1/CD44* in Cancer Types. Cancers 13, 2523. 10.3390/cancers13112523 34063946 PMC8196568

[B58] Molina J. R. Yang P. Cassivi S. D. Schild S. E. Adjei A. A. (2008). Non-small Cell Lung Cancer: Epidemiology, Risk Factors, Treatment, and Survivorship. Mayo Clinic Proc. 83, 584–594. 10.4065/83.5.58410.1016/s0025-6196(11)60735-0 PMC2718421 18452692

[B59] Moliner P. Lupón J. Antonio M. Domingo M. Santiago‐Vacas E. Zamora E. (2019). Trends in Modes of Death in Heart Failure over the Last Two Decades: Less Sudden Death but Cancer Deaths on the Rise. Eur. J. Heart Fail. 21, 1259–1266. 10.1002/ejhf.1569 31359563

[B60] Mulder E. E. A. P. Smit L. Grünhagen D. J. Verhoef C. Sleijfer S. van der Veldt A. A. M. (2021). Cost-effectiveness of Adjuvant Systemic Therapies for Patients with High-Risk Melanoma in Europe: a Model-Based Economic Evaluation. ESMO Open 6, 100303. 10.1016/j.esmoop.2021.100303 34781194 PMC8599106

[B61] Ott J. J. Ullrich A. Miller A. B. (2009). The Importance of Early Symptom Recognition in the Context of Early Detection and Cancer Survival. Eur. J. Cancer 45, 2743–2748. 10.1016/j.ejca.2009.08.009 19765977

[B62] Pan F. Chen Y. He J. Z. Long L. Chen Y. Luo H. J. (2019). Dietary Riboflavin Deficiency Promotes N-Nitrosomethylbenzylamine-Induced Esophageal Tumorigenesis in Rats by Inducing Chronic Inflammation. Am. J. Cancer Res. 9, 2469–2481. 31815047 PMC6895446

[B63] Rathinasamy B. Velmurugan B. K. (2018). Role of lncRNAs in the Cancer Development and Progression and Their Regulation by Various Phytochemicals. Biomed. Pharmacother. 102, 242–248. 10.1016/j.biopha.2018.03.077 29567536

[B64] Saha S. K. Yin Y. Chae H. S. Cho S.-G. (2019). Opposing Regulation of Cancer Properties via KRT19-Mediated Differential Modulation of Wnt/β-Catenin/Notch Signaling in Breast and Colon Cancers. Cancers 11, 99. 10.3390/cancers11010099 PMC6357186 30650643

[B65] Shang Z. Yu J. Sun L. Tian J. Zhu S. Zhang B. (2019). LncRNA PCAT1 Activates AKT and NF-Κb Signaling in Castration-Resistant Prostate Cancer by Regulating the PHLPP/FKBP51/IKKα Complex. Nucleic Acids Res. 47, 4211–4225. 10.1093/nar/gkz108 30773595 PMC6486551

[B66] Shi H. Sun S. Xu H. Zhao Z. Han Z. Jia J. (2020a). Combined Delivery of Temozolomide and siPLK1 Using Targeted Nanoparticles to Enhance Temozolomide Sensitivity in Glioma. Ijn 15, 3347–3362. 10.2147/ijn.s243878 32494134 PMC7229804

[B67] Shi Q. Li Y. Li S. Jin L. Lai H. Wu Y. (2020b). LncRNA DILA1 Inhibits Cyclin D1 Degradation and Contributes to Tamoxifen Resistance in Breast Cancer. Nat. Commun. 11, 5513. 10.1038/s41467-020-19349-w 33139730 PMC7608661

[B68] Shin J. Lim J. S. Huh Y.-M. Kim J.-H. Hyung W. J. Chung J.-J. (2021). A Radiomics-Based Model for Predicting Prognosis of Locally Advanced Gastric Cancer in the Preoperative Setting. Sci. Rep. 11, 1879. 10.1038/s41598-021-81408-z 33479398 PMC7820605

[B69] Sidney S. Go A. S. Rana J. S. (2019). Transition from Heart Disease to Cancer as the Leading Cause of Death in the United States. Ann. Intern. Med. 171, 225. 10.7326/l19-0202 31382279

[B70] Siegel R. L. Miller K. D. Fuchs H. E. Jemal A. (2021). Cancer Statistics, 2021. CA A. Cancer J. Clin. 71, 7–33. 10.3322/caac.21654 33433946

[B71] Singh A. Singh I. Singh N. Puzanov I. (2020). Optimal Management of First-Line Advanced Renal Cell Carcinoma: Focus on Pembrolizumab. Ott 13, 4021–4034. 10.2147/ott.s215173 PMC7231754 32494157

[B72] Sławińska-Brych A. Mizerska-Kowalska M. Król S. K. Stepulak A. Zdzisińska B. (2021). Xanthohumol Impairs the PMA-Driven Invasive Behaviour of Lung Cancer Cell Line A549 and Exerts Anti-EMT Action. Cells 10, 1484. 10.3390/cells10061484 34204745 PMC8231538

[B73] Su M. Yao N. Liu L. Cheng J. Sun X. Yue H. (2021). Older Cancer Survivors Living with Financial Hardship in China: a Qualitative Study of Family Perspectives. Psycho‐Oncology. 10.1002/pon.5854 34784087

[B74] Sun D.-P. Fang C.-L. Chen H.-K. Wen K.-S. Hseu Y.-C. Hung S.-T. (2017). EPAC1 Overexpression Is a Prognostic Marker and its Inhibition Shows Promising Therapeutic Potential for Gastric Cancer. Oncol. Rep. 37, 1953–1960. 10.3892/or.2017.5442 28260059 PMC5367365

[B75] Sun L. Wang L. Chen T. Yao B. Wang Y. Li Q. (2019). microRNA‐1914, Which Is Regulated by lncRNA DUXAP10, Inhibits Cell Proliferation by Targeting the GPR39‐mediated PI3K/AKT/mTOR Pathway in HCC. J. Cel. Mol. Med. 23, 8292–8304. 10.1111/jcmm.14705 PMC6850956 31576658

[B76] Sun Z. Chen T. Thorgeirsson S. S. Zhan Q. Chen J. Park J.-H. (2013). Dramatic Reduction of Liver Cancer Incidence in Young Adults: 28 Year Follow-Up of Etiological Interventions in an Endemic Area of China. Carcinogenesis 34, 1800–1805. 10.1093/carcin/bgt007 23322152 PMC3731800

[B77] Sung H. Ferlay J. Siegel R. L. Laversanne M. Soerjomataram I. Jemal A. (2021). Global Cancer Statistics 2020: GLOBOCAN Estimates of Incidence and Mortality Worldwide for 36 Cancers in 185 Countries. CA A. Cancer J. Clin. 71, 209–249. 10.3322/caac.21660 33538338

[B78] Tan Y. T. Lin J. F. Li T. Li J. J. Xu R. H. Ju H. Q. (2021). LncRNA‐mediated Posttranslational Modifications and Reprogramming of Energy Metabolism in Cancer. Cancer Commun. 41, 109–120. 10.1002/cac2.12108 PMC7896749 33119215

[B79] Tang D. G. (2012). Understanding Cancer Stem Cell Heterogeneity and Plasticity. Cell Res 22, 457–472. 10.1038/cr.2012.13 22357481 PMC3292302

[B80] Wang G. Le Y. Wei L. Cheng L. (2021a). CREB3 Transactivates lncRNA ZFAS1 to Promote Papillary Thyroid Carcinoma Metastasis by Modulating miR-373-3p/MMP3 Regulatory axis. Int. J. Endocrinol. 2021, 1–9. 10.1155/2021/9981683 PMC8238556 34249125

[B81] Wang J. Du S. Wang C. Zhu Z. Xie B. Zhang B. (2021b). Clinicopathological and Prognostic Value of Long Noncoding RNA SNHG7 in Cancers: a Meta-Analysis and Bioinformatics. Aging 13, 23796–23809. 10.18632/aging.203650 34714775 PMC8580357

[B82] Wang J. Wu M. Zheng D. Zhang H. Lv Y. Zhang L. (2020a). Garcinol Inhibits Esophageal Cancer Metastasis by Suppressing the P300 and TGF-Β1 Signaling Pathways. Acta Pharmacol. Sin. 41, 82–92. 10.1038/s41401-019-0271-3 31371781 PMC7471459

[B83] Wang X. F. Chen J. Gong Y. B. Qin Y. C. Wang L. Li N. C. (2019a). Long Non-coding RNA DUXAP10 Promotes the Proliferation, Migration, and Inhibits Apoptosis of Prostate Cancer Cells. Eur. Rev. Med. Pharmacol. Sci. 23, 3699–3708. 10.26355/eurrev_201905_17793 31114994

[B84] Wang Y. Liu J. Bai H. Dang Y. Lv P. Wu S. (2017). Long Intergenic Non-coding RNA 00152 Promotes Renal Cell Carcinoma Progression by Epigenetically Suppressing P16 and Negatively Regulates miR-205. Am. J. Cancer Res. 7, 312–322. 28337379 PMC5336504

[B85] Wang Y. Chen S. Sun S. Liu G. Chen L. Xia Y. (2020b). Wogonin Induces Apoptosis and Reverses Sunitinib Resistance of Renal Cell Carcinoma Cells via Inhibiting CDK4-RB Pathway. Front. Pharmacol. 11, 1152. 10.3389/fphar.2020.01152 32792963 PMC7394056

[B86] Wang Y. Wang W. Wu H. Zhou Y. Qin X. Wang Y. (2021c). The Essential Role of PRAK in Tumor Metastasis and its Therapeutic Potential. Nat. Commun. 12, 1736. 10.1038/s41467-021-21993-9 33741957 PMC7979731

[B87] Wang Y. Yang L. Chen T. Liu X. Guo Y. Zhu Q. (2019b). A Novel lncRNA MCM3AP-AS1 Promotes the Growth of Hepatocellular Carcinoma by Targeting miR-194-5p/FOXA1 axis. Mol. Cancer 18, 28. 10.1186/s12943-019-0957-7 30782188 PMC6381672

[B88] Wang Z. Ren B. Huang J. Yin R. Jiang F. Zhang Q. (2018). LncRNA DUXAP10 Modulates Cell Proliferation in Esophageal Squamous Cell Carcinoma through Epigenetically Silencing P21. Cancer Biol. Ther. 19, 998–1005. 10.1080/15384047.2018.1470723 30215547 PMC6301814

[B89] Wardle J. Robb K. Vernon S. Waller J. (2015). Screening for Prevention and Early Diagnosis of Cancer. Am. Psychol. 70, 119–133. 10.1037/a0037357 25730719

[B90] Wei C.-C. Nie F.-Q. Jiang L.-L. Chen Q.-N. Chen Z.-Y. Chen X. (2017). The Pseudogene DUXAP10 Promotes an Aggressive Phenotype through Binding with LSD1 and Repressing LATS2 and RRAD in Non Small Cell Lung Cancer. Oncotarget 8, 5233–5246. 10.18632/oncotarget.14125 28029651 PMC5354904

[B91] Wen S.-S. Zhang T.-T. Xue D.-X. Wu W.-L. Wang Y.-L. Wang Y. (2019). Metabolic Reprogramming and its Clinical Application in Thyroid Cancer (Review). Oncol. Lett. 18, 1579–1584. 10.3892/ol.2019.10485 31423225 PMC6607326

[B92] Wen S. Wei Y. Zen C. Xiong W. Niu Y. Zhao Y. (2020). Long Non-coding RNA NEAT1 Promotes Bone Metastasis of Prostate Cancer through N6-Methyladenosine. Mol. Cancer 19, 171. 10.1186/s12943-020-01293-4 33308223 PMC7733260

[B93] Wu B. Yang C. Fang Y. Ding W. Zhang Y. (2021). Long Noncoding RNA DUXAP10 Promotes the Stemness of Glioma Cells by Recruiting HuR to Enhance Sox12 mRNA Stability. Environ. Toxicol. 36, 840–849. 10.1002/tox.23087 33340249

[B94] Xu Y. Yu X. Wei C. Nie F. Huang M. Sun M. (2018). Over-expression of Oncigenic Pesudogene DUXAP10 Promotes Cell Proliferation and Invasion by Regulating LATS1 and β-catenin in Gastric Cancer. J. Exp. Clin. Cancer Res. 37, 13. 10.1186/s13046-018-0684-8 29374493 PMC5787324

[B95] Xu Y. Yuan X. Zhang X. Hu W. Wang Z. Yao L. (2021). Prognostic Value of Inflammatory and Nutritional Markers for Hepatocellular Carcinoma. Medicine (Baltimore) 100, e26506. 10.1097/md.0000000000026506 34160470 PMC8238303

[B96] Yao R. Feng W. T. Xu L. J. Zhong X. M. Liu H. Sun Y. (2018). DUXAP10 Regulates Proliferation and Apoptosis of Chronic Myeloid Leukemia via PTEN Pathway. Eur. Rev. Med. Pharmacol. Sci. 22, 4934–4940. 10.26355/eurrev_201808_15632 30070329

[B97] Yi D. Zhang D. He J. (2021). Long Non-coding RNA LIFR-AS1 Suppressed the Proliferation, Angiogenesis, Migration and Invasion of Papillary Thyroid Cancer Cells via the miR-31-5p/SIDT2 axis. Cell Cycle 20, 2619–2637. 10.1080/15384101.2021.1995129 34781815 PMC8726651

[B98] Yin H. Wang X. Zhang X. Wang Y. Zeng Y. Xiong Y. (2018). Integrated Analysis of Long Noncoding RNA Associated-Competing Endogenous RNA as Prognostic Biomarkers in clear Cell Renal Carcinoma. Cancer Sci. 109, 3336–3349. 10.1111/cas.13778 30152187 PMC6172067

[B99] Yu X. Liang C. Zhang Y. Zhang W. Chen H. (2019). Inhibitory Short Peptides Targeting EPS8/ABI1/SOS1 Tri-complex Suppress Invasion and Metastasis of Ovarian Cancer Cells. BMC Cancer 19, 878. 10.1186/s12885-019-6087-1 31488087 PMC6727365

[B100] Zacharias M. Brcic L. Eidenhammer S. Popper H. (2018). Bulk Tumour Cell Migration in Lung Carcinomas Might Be More Common Than Epithelial-Mesenchymal Transition and Be Differently Regulated. BMC Cancer 18, 717. 10.1186/s12885-018-4640-y 29976164 PMC6034257

[B101] Zhang Q. Wang W. W. Xu T. H. Xu Z. F. (2018). Highly Expressed Long Non-coding RNA DUXAP10 Promotes Proliferation of Ovarian Cancer. Eur. Rev. Med. Pharmacol. Sci. 22, 314–321. 10.26355/eurrev_201801_14174 29424918

[B102] Zhang Y. Jia D.-D. Zhang Y.-F. Cheng M.-D. Zhu W.-X. Li P.-F. (2021). The Emerging Function and Clinical Significance of circRNAs in Thyroid Cancer and Autoimmune Thyroid Diseases. Int. J. Biol. Sci. 17, 1731–1741. 10.7150/ijbs.55381 33994857 PMC8120456

[B103] Zhang Z. Zeng K. Zhao S. Zhao Y. Hou X. Luo F. (2019). Pemetrexed/carboplatin Plus Gefitinib as a First-Line Treatment for EGFR-Mutant Advanced Nonsmall Cell Lung Cancer: a Bayesian Network Meta-Analysis. Ther. Adv. Med. Oncol. 11, 175883591989165. 10.1177/1758835919891652 PMC6937538 31908655

[B104] Zhao L. Liu Y. Tong D. Qin Y. Yang J. Xue M. (2017). MeCP2 Promotes Gastric Cancer Progression through Regulating FOXF1/Wnt5a/β-Catenin and MYOD1/Caspase-3 Signaling Pathways. EBioMedicine 16, 87–100. 10.1016/j.ebiom.2017.01.021 28131747 PMC5474507

[B105] Zheng Y. Wu J. Deng R. Lin C. Huang Y. Yang X. (2021). G3BP2 Regulated by the lncRNA LINC01554 Facilitates Esophageal Squamous Cell Carcinoma Metastasis through Stabilizing HDGF Transcript. Oncogene. 10.1038/s41388-021-02073-0 PMC8782723 34782720

[B106] Zhou Q. Liu L. Zhou J. Chen Y. Xie D. Yao Y. (2021). Novel Insights into MALAT1 Function as a MicroRNA Sponge in NSCLC. Front. Oncol. 11, 758653. 10.3389/fonc.2021.758653 34778078 PMC8578859

[B107] Zhu J. Fu H. Wu Y. Zheng X. (2013). Function of lncRNAs and Approaches to lncRNA-Protein Interactions. Sci. China Life Sci. 56, 876–885. 10.1007/s11427-013-4553-6 24091684

[B108] Zhu Q. Liu J. Tang J. Guo D. L. Li Y. Duan R. (2018). Overexpression of Long Non-coding RNAs DUXAP9 and DUXAP10 Is Associated with Prognosis in Patients with Hepatocellular Carcinoma after Hepatectomy. Int. J. Clin. Exp. Pathol. 11, 1407–1414. 31938237 PMC6958151

[B109] Zhu Z. Huang P. Sun R. Li X. Li W. Gong W. (2021). A Novel Long-Noncoding RNA LncZFAS1 Prevents MPP+-Induced Neuroinflammation through MIB1 Activation. Mol. Neurobiol. 10.1007/s12035-021-02619-z PMC8857135 34775541

